# Assessing the Effects of a Perioperative Nutritional Support and Counseling in Gastrointestinal Cancer Patients: A Retrospective Comparative Study with Historical Controls

**DOI:** 10.3390/biomedicines11020609

**Published:** 2023-02-17

**Authors:** Diana Klassen, Carmen Strauch, Birgit Alteheld, Philipp Lingohr, Hanno Matthaei, Tim Vilz, Maria A. Gonzalez-Carmona, Annekristin Hausen, Marie Gräßler, Amit Sharma, Christian Strassburg, Jörg C. Kalff, Ingo G. H. Schmidt-Wolf

**Affiliations:** 1 Center for Integrated Oncology (CIO), Department of Integrated Oncology, University Hospital Bonn, 53127 Bonn, Germany; 2 Department of Nutrition and Food Sciences, Nutritional Physiology, University of Bonn, 53115 Bonn, Germany; 3 Department of General, Abdominal, Thoracic and Vascular Surgery, University Hospital Bonn, 53127 Bonn, Germany; 4 Department of General Internal Medicine, University Hospital Bonn, 53127 Bonn, Germany; 5 Department of Neurosurgery, University Hospital Bonn, 53127 Bonn, Germany

**Keywords:** gastrointestinal cancer, dietary counseling, nutritional support, perioperative nutritional therapy, microbiota

## Abstract

The aim of this study was to investigate the effects of perioperative nutritional therapy care in gastrointestinal (esophageal, gastric, gastroesophageal) cancer patients on nutritional status and disease progression (complications, hospitalization, mortality). We considered 62 gastrointestinal cancer patients treated at the Center for Integrated Oncology (CIO), University Hospital Bonn, Germany (August 2017–July 2019). Of these, 42 patients (as intervention group: IG) received pre- and postoperative nutritional support with counseling, while 20 patients (as historical control group CG) received only postoperative nutritional therapy. Several clinical parameters, such as Body Mass Index (BMI), nutritional risk screening (NRS), phase angle, postoperative complications, length of hospital stay, and mortality, were determined. There were significantly fewer patients with gastric cancer/*CDH1* gene mutation and more with esophageal cancer in IG (*p* = 0.001). Significantly more patients received neoadjuvant therapy in IG (*p* = 0.036). No significant differences were found between the groups regarding BMI, NRS, complications, length of hospital stay, and mortality. However, the comparison of post- and preoperative parameters in IG showed a tendency to lose 1.74 kg of weight (*p* = 0.046), a decrease in phase angle by 0.59° (*p* = 0.004), and an increase in NRS of 1.34 points (*p* < 0.001). Contrary to prior reports, we found no significant effect of perioperative nutritional therapy care in gastrointestinal cancer patients; however, the small cohort size and infrequent standardization in nutritional status may possibly account for the variance. Considering that oncological pathways and metabolic nutritional pathways are interrelated, dividing patients into subgroups to provide a personalized nutritional approach may help in improving their treatment.

## 1. Introduction

Tumor-associated malnutrition is a major problem in oncology that is often inadequately addressed in the therapeutic regimens of affected patients. Malnutrition is caused by cancer itself and the tumor-associated treatment (surgery, chemotherapy, or radiotherapy), as well as by lack of nutritional therapeutic measures. It is now well established that maintaining body weight can help cancer patients not only avoid the adverse effects of treatment therapies, but also improve their survival rate. At the same time, it cannot be completely ignored that cancer and cancer therapies independently lead to metabolic derailments varying from loss of appetite (malnutrition) to a highly complex cachexia state (systemic inflammation, unfavorable protein and energy balance, loss of lean body mass) in patients. Considering this, the German Society for Nutritional Medicine (DGEM) and the European Society for Clinical Nutrition and Metabolism (ESPEN) have recommended updated guidelines to improve body resources, tolerability of cancer treatments, overall prognosis, and follow-up in terms of patient quality [1,2].

Notably, gastrointestinal (GI) cancers often remain in the focus, primarily due to the higher risk of malnutrition owing to poor digestion, malabsorption caused by a blocked GI tract, and therapies such as surgical resection and chemotherapy. After surgical treatment, patients have a reduced food intake and complications, such as inadequate absorption of nutrients, and intolerance can occur [3,4,5,6]. The goals of nutritional therapy for cancer patients include not only preventing weight loss and malnutrition, but also improved and individually adapted dietary intake and reduction of metabolic disorders. There have been reports that patients with gastric and esophageal cancer have significant weight loss at the time of diagnosis, which continues to advance during therapy [7,8,9]. Rosania and colleagues showed that preoperative nutritional status directly affects postoperative prognosis in gastric cancer (GC) patients; also, perioperative nutritional support reduces overall complications, but not mortality [10]. Whether or not oral nutritional treatment provides any clinical benefits for long-term oncological outcomes in GI remains unclear, but several studies continue to evaluate the effects of different nutritional interventions (ONS: oral nutritional supplements, EN: enteral nutrition, PN: parenteral nutrition) in cancer patients (e.g., gastric cancer) [11]. Interestingly, a few studies have investigated the influence of nutritional support (EN versus PN) on gastrointestinal microbiota [12], and the link between gastric microbiota and gastric cancer is certainly an emerging area of research [13,14].

Considering that adequate nutritional intake can improve chemotherapy tolerance and survival [15], it has been demonstrated that stable weight compared with weight loss or preoperative cachexia in patients with gastrointestinal cancer leads to a significant increase in survival, quality of life, and shorter hospital stay [16,17]. Here, we investigated the effects of perioperative nutritional therapy care in gastrointestinal (esophageal, gastric, gastroesophageal) cancer patients treated at the Center for Integrated Oncology (CIO), University Hospital Bonn, Germany (August 2017–July 2019). In this single-center study, we classified all the clinically well-defined patient groups and measured several outcome and medical parameters (body mass index, nutritional risk screening, phase angle, postoperative complications, length of hospital stay, and mortality).

## 2. Materials and Methods

### 2.1. Patients

All enrolled patients (*n* = 62) were diagnosed with GI cancer (esophageal cancer, gastroesophageal junction cancer, gastric cancer) and were treated (including surgery and nutritional support by a dietitian) at the CIO, University Hospital Bonn, Germany (August 2017–July 2019). The diagnosis was made using the TNM classification with a cancer staging process according to the Union for International Cancer Control (UICC) [18,19]. Patients with CDH1 gene mutation (risk factor for gastric cancer) and gastrectomy were also specified. Notably, due to the reorganization in standard nutritional therapy care from 2017 to 2019, patients initially received nutritional therapy only postoperatively, while preoperative nutritional therapy was later implemented into the standard care. For comprehensive analysis, patients were classified into an intervention group (IG, *n* = 42), which received preoperative and postoperative nutritional support with counseling, and a historical control group (CG, *n* = 20), which received only postoperative nutritional therapy (as they were treated before the reorganization of the standard care). The detailed clinical characteristics of the study population are presented in Table 1.

### 2.2. Study Design

In this single-center retrospective study, the nutritional status and multiple clinical parameters, such as nutritional risk screening (NRS), bioelectrical impedance analysis (BIA) phase angle, weight loss, postoperative complications, lengths of hospital stay, and mortality, were investigated. Specifically, changes in NRS, phase angle, and weight within IG were examined pre- and postoperatively. Other required information was extracted from patients’ electronic health records.

### 2.3. Nutritional Therapy

IG patients were consulted (at least once pre- and postoperatively) for detailed nutritional counseling, which lasted approximately 45 min. However, a few additional counseling sessions were also provided at the request of physicians. Nutritional history, including anthropometric data, such as weight, disease-related weight loss, and height, was obtained by the dietitian. Quantitative and qualitative food intake, appetite, and gastrointestinal symptoms were also asked. In case of persistent nutritional problems, such as low food intake or malnutrition, high-caloric fluid supplements (2–3 potions (200 mL) per day with 2.0 kcal/mL) and enteral or parenteral nutrition were prescribed. In addition to the changes in nutritional physiology and/or possible complications from the surgery, some practical recommendations to avoid gastrointestinal symptoms, the risk of malnutrition, possible symptoms of dumping syndrome, the necessity of pancreatic enzyme supplementation, and possible lactose intolerance were also discussed. We also calculated the energy requirements and derived a recommendation for protein intake, weight maintenance, and weight gain, respectively. The total energy expenditure was calculated as 25–30 kcal/kg body weight per day, depending on patient activity, and recommended protein intake was calculated as 1.2–1.5 g/kg body weight per day, according to DGEM and ESPEN guidelines [1,2]. Additionally, each patient received written nutritional recommendations related to surgery or individual symptoms, and the contents of the nutritional therapy were documented in the electronic medical record. A standard stepwise introduction to a full diet was further provided to the patients in accordance with the Enhanced Recovery after Surgery (ERAS) Standard Operating Procedure (SOP). All patients received supportive parenteral nutrition (SMOF lipid at 2200 kcal/d) for at least the first 4 postoperative days. All parts of nutritional therapy followed the recommendations of standardized clinical practice guidelines from DGEM and ESPEN. It is worth mentioning that the postoperative nutritional treatment was comparable in both study groups. 

### 2.4. Outcome Parameters

The outcome parameters (weight, NRS, phase angle) on the nutritional status were collected during dietary counseling by the nutritionist. Weight was measured with a calibrated body scale (seca 769), and patients wore light clothing. Phase angle, as a marker for the quality of the muscle mass, was collected by bioelectrical impedance analysis. For this purpose, the multifrequency impedance analyzer Nutriguard-MS Version 2 (Data Input GmbH; Pöcking, Germany) was used. The implementation of the BIA measurement was based on the specifications of Data Input GmbH and official guidelines [20]. It is worth mentioning that the NRS 2002 serves as an evaluated and validated screening tool to determine the risk of malnutrition, taking into account nutritional history (food intake, current weight, weight loss) and disease severity and higher risk in elderly patients [21,22]. It is important to mention that nutritional counseling was irregular in the preoperative and postoperative periods, so the parameters were not standardized over time. As CGs did not receive preoperative nutritional counseling, their outcome parameters on nutritional status were omitted. Instead, the postoperative parameters were compared between the study groups. The medical parameters used to assess the effects of nutritional therapy include postoperative complications, hospitalization, and mortality. The postoperative complications were evaluated using the Clavien–Dindo classification, and a score of IIIb and/or higher was classified as severe complication. The parameter for hospital stay was subdivided into different levels of care (ICU: intensive care unit, MCU: medium care unit, normal ward). The NRS was used to combine the severity of the disease with the nutritional status.

### 2.5. Statistical Analysis

All statistical analyses were performed using IBM SPSS Statistics for Windows (Version 25.0. Armonk, NY, USA: IBM Corp.), and a *p*-value < 0.05 was considered statistically significant. Changes in NRS, phase angle, and weight within IG at two different time points (preoperative and postoperative) were determined using the paired-samples T test, and the results are presented as mean ± standard deviation and as difference in mean ± difference in standard deviations. To compare the outcome parameters of IG and CG, metric data are presented as mean ± standard deviation; also, hospitalization is indicated as median values and is analyzed with the Mann–Whitney U test (no normal distribution was given). The categorical variables are presented as absolute frequencies and relative frequencies in percentages. The categorical data with the condition of an expected cell count <5 in no more than 20% of the counts were analyzed with Pearson’s chi square test (gender, treatment, postoperative complications (yes/no), NRS) [23]. In case of more than 20% of the cells with an expected cell count <5 (postoperative complications scored by Clavien-Dindo, disease stage, diagnosis, mortality, and postoperative BMI), the Fisher exact test was used.

## 3. Results

### 3.1. Patients’ Characteristics

As shown in Table 1, patients with gastrointestinal cancer were divided into subgroups (esophageal cancer, gastroesophageal junction cancer, gastric cancer, *CDH1* gene mutation), and analysis was performed by comparing two groups (IG versus CG). No significant gender difference was observed between these two groups. Similarly, in the context of age, the initial diagnosis was made at an age of 59 ± 13 years, and no statistically significant age-related difference was found. Interestingly, the overall distribution of diagnoses differed significantly between these groups (*p* = 0.001). Since mutation or transcriptional silencing of the *CDH1* gene is associated with gastric cancer, we also screened for the presence/absence of the mutation in this particular gene. Of the 33.3% of IGs diagnosed with gastric cancer in our cohort, only 4.8% were found to be harboring mutation in the *CDH1* gene, whereas this ratio was comparatively high in the case of CGs (55% diagnosed with gastric cancer, 30% carrying a mutation in the *CDH1* gene). It is worth mentioning that the number of patients was generally predominating in IG (esophageal cancer—IG: 26.2%, CG: 10%; gastroesophageal junction cancer—IG: 35.7%, CG: 5%). The majority of the study population was at stage I (41.7%), followed by stage II (26.7%), stage III (18.3%), and stage IV (8.3%). Notably, there was one missing value for disease stage in IG and CG. Here again, no significant difference was observed between these groups at different stages of the disease according to the Union for International Cancer Control (UICC). While CG had resective surgery within 52 ± 54 days of diagnosis, IG underwent surgery within 93 ± 40 days of diagnosis (*p* = 0.009). This is also attributed to the more time IG received for further neoadjuvant therapies compared with CG (IG: 73.8%, CG: 40% (*p* = 0.036)). Overall, no significant differences in adjuvant therapy were found when comparing the two groups. 

### 3.2. Evaluation of Pre- and Postoperative Nutritional Consultation in IG and CG

**Figure 1:**
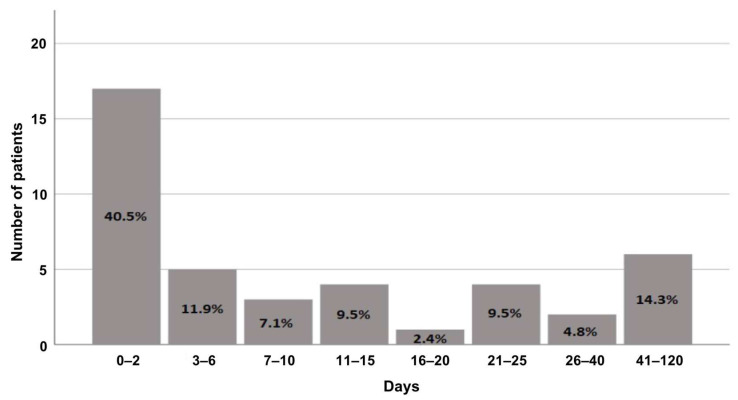
Time between preoperative nutritional consultation and surgery.

**Figure 2:**
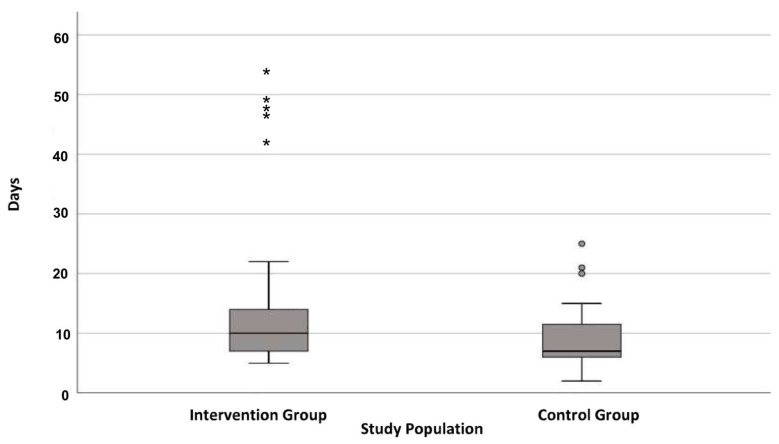
Boxplot on days between surgery and postoperative nutritional consultation. 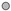 outlier, * extreme outlier.

Of the patients, 40.5% received preoperative nutritional counseling within 2 days before surgery (Figure 1). Overall, preoperative consultation took place at an average of 22 days before surgery (median = 6). Concerning the timing of postoperative counseling, in IG, nutritional counseling was provided on average 15 days after surgery, whereas in CG, it occurred much earlier, that is, on the ninth postoperative day (Figure 2). However, no significant difference was observed in a median (MD) comparison (MD: IG = 10; CG = 7). The same scenario was observed in a long range (approx. 60 days) of nutritional consultation. Notably, a few patients were identified as statistical outliers in both groups (IG: *n* = 5, 49 days; CG: *n* = 3, 23 days) because they received postoperative nutrition counseling considerably later. Here, it is important to mention that counseling was performed on an individual basis, when the patients were receptive and had made sufficient recovery following surgery. Since the duration of the gradual transition to a full diet was highly personalized, there were some patients who needed counseling earlier than others. This led to minor disparity between the IG and CG groups.

### 3.3. Comparison of Pre- and Postoperative Parameters in IG 

The average time between pre- and postoperative nutritional counseling was 37 ± 35 days with a range of 119 days and a median of 21 days. Among the four parameters considered (NRS, weight, BMI, phase angle), a significant postoperative increase in NRS of 1.34 points (*p* < 0.001) was observed (Table 2). As compared with preoperative (mean score of 2.37), postoperative (mean score of 3.71) were found to be associated with an increased risk of malnutrition (≥3 points). It is worth mentioning that all patients were generally at risk of malnutrition after surgery, but a subset of patients (45.2%) was at high risk of malnutrition before surgery (more than 2 points). A trend of minor differences was also observed in the case of weight; for instance, the proportion of patients who were underweight preoperatively was 2.4%, which increased to 4.9% after surgery. Perioperative weight loss was on average 1.74 kg (*p* = 0.046). It is worth mentioning that, according to NRS prescreening, 9.5% of the patients had critical BMI (<20.5 kg/m²) before surgery, whereas postsurgery, this number rose to 12.2%. Although perioperative weight loss was significant, a decrease in BMI of 0.5 kg/m² was not significant.

The phase angle, an additional parameter we used to assess the quality of muscle mass, was already too low before the surgery (mean value of 4.83°, ideal value ≥ 5.0°) and further decreased perioperatively by 0.59° (*p* = 0.004). Overall, 44.1% of patients had a reduced phase angle before surgery compared with 72.7% postoperatively. 

### 3.4. Comparison of Outcomes between IG and CG

**Figure 3:**
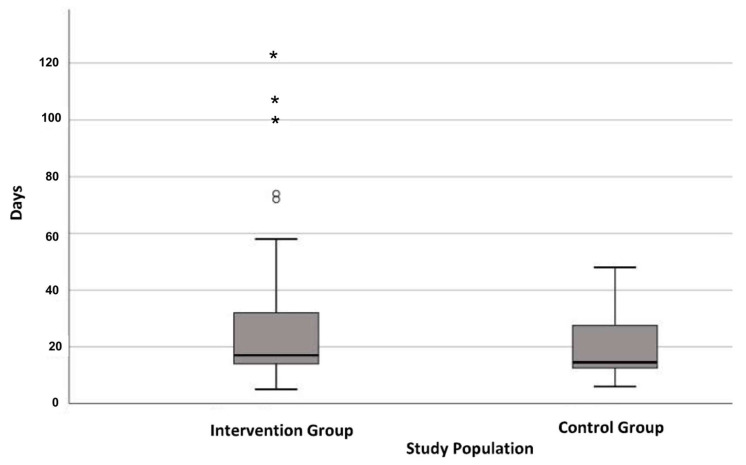
Boxplot on total length of hospital stay during surgery. ° outlier, * extreme outlier.

No difference was observed in total length of hospital stay (ICU, MCU, normal ward) in both groups, and the majority of patients showed quite a similar pattern (IG: 14–35 days, CG: 12–28 days) (Figure 3, Table 3). For instance, IG spent 9 ± 19 days in ICU, while CG was only for 2 ± 3 days, but the median of these two in ICU did not differ between groups (*p* = 0.735).

Likewise, no significant differences were observed in the number of patients with complications and/or with severe complications in these groups. A slight difference in complications that occurred in the IG group may be due to differences in diagnosis (fewer *CDH1* gene mutations) and higher overall risk. In addition, significantly more patients in the IG group received neoadjuvant treatment. Nevertheless, the differences in complications between the two groups were not found to be significant. Overall mortality was found to be 6.5%, but without any statistical significance. Postoperatively, all the patients had NRS of at least 3, indicating increased nutritional risk, with the highest score achieved being 5. The distribution of the BMI was also found to be similar in both groups. The majority of patients had normal weight (44.3%) or were overweight (32.8%), and a few were obese (18%) or underweight (4.9%).

## 4. Discussion

Although a positive effect of nutritional therapy care can be assumed in oncological and surgical patients, there are only a few studies that have investigated or been able to show this. Here, we investigated the effect of perioperative nutritional support and counselling on various outcome parameters in patients with gastrointestinal (esophageal, gastric, gastroesophageal) cancers. In this single-center study, we found no significant differences between groups concerning BMI, NRS, complications, length of hospital stay, and mortality. However, the comparison of post- and preoperative parameters in IG showed a tendency to lose 1.74 kg of weight (*p* = 0.046), a decrease in phase angle by 0.59° (*p* = 0.004), and an increase in NRS of 1.34 points (*p* < 0.001).

Cancer, being a complex and heterogeneous disease [24,25], completely relies on the efficiency of cancer therapies, which broadly not only affect cancer cells, but also have a strong toxic effect on neighboring healthy tissues. Besides, the altered metabolism due to the cancer itself or malfunctioning of the organ affected by the cancer can easily lead to a state of malnutrition or weakness, which further complicates the therapeutic regimen. To avoid such malnutrition related complications, nutritional oncology acts as a multidisciplinary approach to support the performance of traditional therapies (chemotherapy and radiotherapy) and enhances the patient’s survival rate. The necessity of nutritional therapy/counseling can be evident from the fact that if not timely (preoperative and postoperative) provided, the patient’s malnutrition state (e.g., weight loss) may lead to clinical challenges, such as early termination of ongoing therapy. Some studies have linked nutritional status with favorable outcome measures, such as decrease in length of hospital stay, reduced postoperative complications, and improvements in the quality of life [10,16,17,26,27,28,29]. A few others have shown an association of nutritional status with weight loss, low BMI, and high postoperative mortality [30,31]. Notably, gastrointestinal (GI) cancers often have additional concerns, first, due to higher risk of malnutrition owing to poor digestion, and second, the tumor progression blocks the areas of the GI tract to interrupt the nutrient absorption. Therefore, the notion of nutritional support in GI cancers always remains a major area of interest [10,32,33].

So far, few studies have investigated not only nutritional status but also the effects of nutritional therapeutic care or counseling on the outcome. For instance, one study showed that even a fact sheet with nutritional information could improve the lack of knowledge of patients with breast cancer [34]. Another study that examined the effects of intensive perioperative nutritional therapy care as part of multidisciplinary management (in surgical patients with esophageal cancer) revealed that in the absence of such approach, higher weight loss and more frequent postoperative complications can be expected [35]. However, in our present study, no significant results were observed. Since most studies focus mainly on nutritional status or enteral/parenteral nutrition, the effects of nutritional counseling are often ignored. However, we have incorporated all in-depth information on this issue. The insufficient awareness of the impact of malnutrition in the medical community is worth mentioning. Thus, our study underlines the importance of nutritional therapy as part of cancer treatment and the work of interprofessional nutrition teams. Another key strength of the study involves the standardized nutritional therapy and counseling according to DGEM and ESPEN guidelines [1,2] and the Enhanced Recovery after Surgery (ERAS) Standard Operating Procedure (SOP), which was similar in both groups. It is important to mention the limitations of our study, such as (1) the timing of nutritional counseling was not standardized; thus the assessment of anthropometric parameters displayed variations in some cases. (2) The lack of comparable CG, as our retrospective study used only a historical CG, where patients were treated before the implementation of perioperative nutritional therapy. (3) The small cohort size, as subgroup analysis could not be performed because of the small numbers of cases within each diagnosis. As previously mentioned, counseling was performed on an individual basis, when the patients were receptive and had made sufficient recovery following surgery. This also led to some differences between the IG and CG groups.

However, when comparing our data especially for IGs with other studies, we obtained quite similar conclusions. For instance, the percentage of patients in the IG who had an increased risk of malnutrition preoperatively was comparable to other studies [36,37]. The average perioperative weight loss of 2.16% within 5 weeks in our study was even lower compared with an independent study involving a multidisciplinary nutrition team (weight loss of 6.4% with nutritional care compared with 10.3% without intensive care) [35]. Notably, the variation in low phase angle that we observed was also previously considered to be an independent risk factor for postoperative complications [38], and even a phase angle of less than 4.8° was found to be linked with shorter survival in hepatocellular carcinoma [26]. One study showed that intensive nutritional support from a nutritionist was associated with a significant reduction in severe postoperative complications in patients with esophageal cancer [39]. Compared with the above-mentioned study, the patients in the present study received less intensive preoperative care, but it is still noticeable that the patients experienced a lower complication rate and a shorter length of hospital stay. Based on our observations, it is reasonable to assume that preoperative care may require more attention. Several other studies have also pointed out that intensive preoperative care can significantly improve patient outcomes [35,39]. However, stringent methodological criteria are required to clearly distinguish the benefits of preoperative versus postoperative nutritional interventions.

On a future prospective of such nutritional oncology studies, we would like to point out some early studies that have investigated the impact of nutritional support (EN versus PN) on the gastrointestinal microbiota [12]. Since the relationship between gastric microbiota and gastric cancer has already been pointed out [13,14], we therefore suggest to enhance translational studies using a personalized nutritional approach to connect gastrointestinal microbiota and inter-/intraindividual patient therapeutic variability. As systemic inflammation is another important factor influencing the clinical outcome of patients, it would also be beneficial to consider this parameter in future studies. Future studies in this area should focus also on intensive nutritional care preoperatively with postoperative follow-up to ensure positive effects on treatment outcome. This could help to improve the nutritional counseling, therapy, and outcome of cancer patients. Considering the differences in diagnosis between the groups in our study, future studies should focus only on one tumor entity to gain better results. Given that only a few studies examine the cumulative effect of nutritional status and nutritional counseling in cancers, more randomized intervention trials should be conducted to gain deeper insights into their utility for standard patient care.

## 5. Conclusions

Contrary to prior reports, we found no significant effects of perioperative nutritional therapy care in gastrointestinal cancer patients; however, the small cohort size and infrequent standardization (immediate/long-term assessment) in nutritional status may possibly account for the variance in our single-center study. Considering that oncological pathways and metabolic nutritional pathways are interrelated, dividing patients into subgroups primarily based on the personalized nutritional approach may help in improving their treatment. Intensive preoperative dietary intervention may also improve the nutritional state of patients undergoing surgery and should be considered in further studies.

## Figures and Tables

**Table 1:** Characteristics of the study population.

	All Patients (*n* = 62)	Intervention Group (*n* = 42)	Control Group (*n* = 20)	*p*-Value *
Gender (m/f)	40/22	25/17	15/5	0.234
Age at time of diagnosis ^1^	59 ± 13	61 ± 11	54 ± 17	0.219
**Diagnosis, *n* (%) ^2^**				0.001
Esophageal cancer	13 (21.0)	11 (26.2)	2 (10,0)	
Cancer of the	16 (25.8)	15 (35.7)	1 (5.0)	
gastroesophageal junction				
Gastric cancer	25 (40.3	14 (33.3)	11 (55.0)	
***CDH1*** gene mutation	8 (12.9)	2 (4.8)	6 (30.0)	
**UICC stage of disease, *n* (%) ^2^**				
0	3 (5.0)	3 (7.3)	0 (0.0)	
I	25 (41.7)	13 (31.7)	12 (63.2)	
II	16 (26.7)	14 (34.1)	2 (10.5)	0.114
III	11 (18.3)	8 (19.5)	3 (15.8)	
IV	5 (8.3)	2 (10.5)	2 (10.5)	
**Time between diagnosis and surgery (days)^1^**	80 ± 49	93 ± 40	52 ± 54	0.009
**Treatment before surgery, *n* (%) ^2^**				0.036
No treatment	23 (37.1)	11 (26.2)	12 (60.0)	
Chemotherapy	30 (48.4)	24 (57.1)	6 (30.0)	
Radiochemotherapy	9 (14.5)	7 (16.7)	2 (10.0)	
**Treatment after surgery, *n* (%) ^2^**				
Chemotherapy	19 (30.6)	15 (35.7)	4 (20.0)	0.210

^1^ Data presented as mean ± standard deviation (SD), ^2^ data presented as absolute frequency (relative frequency in %) * Significance level *p* ≤ 0.05.

**Table 2:** Comparison between pre- and postoperative parameters of the Intervention Group.

	MEAN ± SD Preoperative	MEAN ± SD Postoperative	Difference in Mean ± SD of Difference in Means	*p*-Value *
**NRS**	2.37 ± 0.92	3.71 ± 0.64	−1.34 ± 0.97	<0.001
**Weight**	80.60 ± 19.06	78.86 ± 18.03	1.74 ± 5.42	0.046
**BMI**	26.7 ± 5.6	26.2 ± 5.5	0.5 ± 1.7	0.052
**Phase angle**	4.83 ± 0.94	4.24 ± 1.16	0.59 ± 0.80	0.004

standard deviation (SD); * Significance level *p* ≤ 0.05.

**Table 3:** Comparison of postoperative outcomes between intervention group and control group.

	All Patients (*n* = 62)		Intervention Group (*n* = 42)		Control Group (*n* = 20)		*p*-Value *
**Hospital stay**	**MEAN ± SD**	**MD**	**MEAN ± SD**	**MD**	**MEAN ± SD**	**MD**	**MD**
Total	27 ± 24	16	30 ± 28	17	20 ± 11	15	0.237
ICU	7 ± 16	2	9 ± 19	2	2 ± 3	2	0.735
MCU	3 ± 5	2	4 ± 5	2	3 ± 5	0	0.642
Normal ward	16 ± 15	12	18 ± 18	12	14 ± 8	13	0.937
**Complications ^†,1^**	24 (38.7)		19 (45.2)		5 (25.0)		0.126
**Complications scored by Clavien–Dindo ^†,1^**							0.105
No complication	38 (61.3)		23 (54.8)		15 (75.0)		
Grade I	7 (11.3)		7 (16.7)		0 (0.0)		
Grade II	2 (3.2)		1 (2.4)		1 (5.0)		
Grade IIIa	9 (14.5)		5 (11.9)		4 (20.0)		
Grade IIIb	1 (1.6)		1 (2.4)		0 (0.0)		
Grade IVa	5 (8.1)		5 (11.9)		0 (0.0)		
Grade IVb	0 (0.0)		0 (0.0)		0 (0.0)		
Grade V	0 (0.0)		0 (0.0)		0 (0.0)		
**Severe compl.,**	6 (9.7)		6 (14.3)		0 (0.0)		0.164
(≥ IIIa) 1							
Mortality ^1^	4 (6.5)		3 (7.1)		1 (5.0)		0.612
**NRS ^†,1^**							0.538
3	23 (37.7)		16 (39.0)		7 (35.0)		
4	30 (49.2)		21 (51.2)		9 (45.0)		
5	8 (13.1)		4 (9.8)		4 (20.0)		
**BMI ^†,1^**							0.915
Underweight	3 (4.9)		2 (4.9)		1 (5.0)		
Normal weight	27 (44.3)		17 (41.5)		10 (50.0)		
Overweight	20 (32.8)		14 (34.1)		6 (30.0)		
Obese	11 (18.0)		8 (19.5)		3 (15.0)		

SD, standard deviation; MD, median; ^1^, data presented as absolute frequency (relative frequency in %); ^†^, postoperative, * Significance level *p* ≤ 0.05.

## Data Availability

The datasets used and/or analyzed during the current study are available upon request from the corresponding author.
